# A CD44^high^/EGFR^low^ Subpopulation within Head and Neck Cancer Cell Lines Shows an Epithelial-Mesenchymal Transition Phenotype and Resistance to Treatment

**DOI:** 10.1371/journal.pone.0044071

**Published:** 2012-09-25

**Authors:** Linnea La Fleur, Ann-Charlotte Johansson, Karin Roberg

**Affiliations:** 1 Division of Oto-Rhino-Laryngology and Head & Neck Surgery, Department of Clinical and Experimental Medicine, Faculty of Health Sciences, Linköping University, Linköping, Sweden; 2 Department of ENT – Head and Neck Surgery, UHL, County Council of Östergötland, Linköping, Sweden; Virginia Commonwealth University, United States of America

## Abstract

Mortality in head and neck squamous cell carcinoma (HNSCC) is high due to emergence of therapy resistance which results in local and regional recurrences that may have their origin in resistant cancer stem cells (CSCs) or cells with an epithelial-mesenchymal transition (EMT) phenotype. In the present study, we investigate the possibility of using the cell surface expression of CD44 and epidermal growth factor receptor (EGFR), both of which have been used as stem cell markers, to identify subpopulations within HNSCC cell lines that differ with respect to phenotype and treatment sensitivity. Three subpopulations, consisting of CD44^high^/EGFR^low^, CD44^high^/EGFR^high^ and CD44^low^ cells, respectively, were collected by fluorescence-activated cell sorting. The CD44^high^/EGFR^low^ population showed a spindle-shaped EMT-like morphology, while the CD44^low^ population was dominated by cobblestone-shaped cells. The CD44^high^/EGFR^low^ population was enriched with cells in G0/G1 and showed a relatively low proliferation rate and a high plating efficiency. Using a real time PCR array, 27 genes, of which 14 were related to an EMT phenotype and two with stemness, were found to be differentially expressed in CD44^high^/EGFR^low^ cells in comparison to CD44^low^ cells. Moreover, CD44^high^/EGFR^low^ cells showed a low sensitivity to radiation, cisplatin, cetuximab and gefitinib, and a high sensitivity to dasatinib relative to its CD44^high^/EGFR^high^ and CD44^low^ counterparts. In conclusion, our results show that the combination of CD44 (high) and EGFR (low) cell surface expression can be used to identify a treatment resistant subpopulation with an EMT phenotype in HNSCC cell lines.

## Introduction

Head and neck cancer is a malignancy that despite advances in therapy still is associated with severe mortality. Mortality remains high due to emergence of local and regional recurrences and development of lymph node metastasis and, occasionally, distant metastasis. The standard treatment for head and neck squamous cell carcinoma (HNSCC) patients is radiotherapy, often in combination with surgery. Chemoradiotherapy has recently become part of the treatment of advanced tumors, and cisplatin is the most common agent in combination with radiation. In recent years, inhibition of epidermal growth factor receptor (EGFR) signaling by use of anti-EGFR antibodies (e.g., cetuximab and panitumumab) or EGFR tyrosine kinase inhibitors (e.g., gefitinib and erlotinib) has emerged as a new treatment strategy; however, so far cetuximab is the only EGFR-targeted drug approved for the treatment of HNSCC. Radio- and/or chemotherapy resistance and tumor recurrences are important clinical problems in the management of HNSCCs and the need for more effective treatment strategies is urgent [1].

HNSCC, among other epithelial cancers, is a highly heterogeneous disease [2], [3]. It has been known for a long time that there are subpopulations of cells within a tumor that differ from each other with regard to tumorigenicity and metastatic potential [4]–[6]. Recurrent tumors are often therapy-resistant and may have their origin in resistant cancer stem cells (CSCs) or in tumor cells with an epithelial-mesenchymal transition (EMT) phenotype. EMT is a process whereby epithelial cells lose their polarity and cell-cell contact and acquire a migratory mesenchymal phenotype and it has been shown that EMT could promote stem cell properties. Furthermore, the *in vitro* sensitivity of HNSCC cell lines to anticancer agents, including EGFR inhibitors and cisplatin, has been shown to be influenced by the expression of EMT-associated genes [7], [8]. In HNSCC [9] as well as in many other cancers e.g., brain, prostate, lung, colon, pancreas, liver, melanoma and skin neoplasms [10]–[15], presence of CSCs have been reported. Prince et al showed that CD44+, but not CD44-, cells isolated from primary HNSCC samples could give rise to tumors in mice [16]. A high cell surface expression of CD44 has also been used as a marker for CSCs in HNSCC cell lines [17], [18]. Recently it was published that the EGFR was not present on the surface of normal and cancer keratinocytes satisfying the criteria for stem cells, such as quiescence, sphere formation ability and expression of stem cell markers, while keratinocytes displaying EGFR had a more differentiated phenotype [19]. This suggests that a low EGFR cell surface expression can be used as a marker for CSCs in epithelial tumors.

Most traditional cancer treatments do not take into consideration the possibility that subpopulations of cancer cells exist and that these may experience varying sensitivity towards a particular treatment. Indeed, leukemic stem cells have been shown to be more resistant to standard cytotoxic treatments [20], [21] and both glioblastoma stem cells and breast cancer-initiating cells are more radioresistant [22], [23].

The present study investigates the existence and properties of subpopulations of cells in three HNSCC cell lines, LK0827, LK0863 and LK0923. Based on the cell surface expression of CD44 and EGFR, three populations were identified and analyzed with regard to CSC and EMT characteristics as well as radio-, cisplatin, cetuximab, gefitinib and dasatinib (multi-BCR/ABL and Src family tyrosine kinase inhibitor) sensitivity.

## Materials and Methods

### Ethics statement

Fresh HNSCC tumor biopsies were sampled in the tumor collection (No 416, The national board of health and welfare in Sweden) at the Department of ENT – Head and Neck Surgery, Linköping University Hospital (approved by the Regional Ethical Review Board at Linköping University). Cultures used in this study (LK0923, LK0827 and LK0863) were derived from this collection and in accordance with the ethics board. A written informed consent has been obtained from all participants involved in this study. All data analysis associated with these cultures was performed anonymously.

### Cells and culture conditions

The HNSCC cell lines LK0923 (larynx cancer), LK0827 (tongue cancer) and LK0863 (larynx cancer), were established from fresh HNSCC tumor samples at the Department of ENT – Head and Neck Surgery, Linköping University Hospital as previously described [24]. Immediately after excision, tumor samples were immersed in Ca^2+^- and Mg^2+^-free phosphate-buffered saline (PBS-A). The tissue was washed, minced into 1 to 2 mm pieces and placed in culture flasks for propagation. Thereafter, growth medium (Keratinocyte-SFM; GIBCO, Invitrogen Corporation, Paisley, UK) supplemented with antibiotics (penicillin 50 IU/ml, streptomycin 50 µg/ml), fungizone (0.25 µg/ml) and 20% fetal bovine serum (FBS; all from GIBCO) was added and the tumor explants were incubated at 37°C in 5% CO_2_ humidified air. Fibroblasts contaminating the cultures were continuously removed by differential trypsinization (0.25% trypsin +0.02% EDTA [ethylenediaminetetraacetic acid]). Established cell cultures were grown in Keratinocyte-SFM supplemented with antibiotics and 1% FBS, given fresh culture media twice a week and subcultured at confluence using 0.25% trypsin +0.02% EDTA with a weekly split ratio of approximately 1∶2. Cells were screened periodically for mycoplasma contamination using DAPI staining and/or the Universal Mycoplasma Detection Kit (ATCC, Manassas, VA, USA). Cultures in passages 8 to 15 were used in the experiments.

### Flow cytometry and fluorescence-activated cell sorting (FACS)

Cells were detached using Accutase (PAA Laboratories, Pasching, Austria) and subjected to direct immunofluorescence staining. Cells were incubated in 10% FBS in PBS-A at 4°C, thereafter washed in PBS-A with 1% FBS and incubated with antibodies towards CD44 (APC-CD44; Becton Dickinson, San José, CA, USA; 1∶10) and EGFR (PE-EGFR; Abcam, Cambridge, MA, USA; 1∶10) or isotype control antibodies (APC mouse IgG2b; Becton Dickinson and PE rat IgG2a; eBioscience, San Diego, CA, USA, respectively). Cells were washed, suspended in PBS-A and passed through 50 μm filters (Becton Dickinson). Samples were analyzed on a FACS Aria (Becton Dickinson, FACS Aria special order system) and cells with high CD44 and low EGFR cell surface expression (CD44^high^/EGFR^low^), cells with high expression of both markers (CD44^high^/ EGFR^high^), and cells with a low CD44 surface expression (CD44^low^) were collected.

### Proliferation assay and treatment sensitivity

Immediately after cell sorting by FACS, the subpopulations were seeded in 12-well plates. After 10 days of culture cells were fixed in 4% paraformaldehyde (PFA), stained with crystal violet (0.04% in 1% ethanol) and thereafter solubilized in 1% sodium dodecyl sulphate (SDS). The optical density at 550 nm was measured using a Victor plate reader (EG & G Wallac, Upplands Väsby, Sweden).

For determination of treatment response, cells were seeded into 12-well plates and after 24 h irradiated (4 Gy) with 4MeV photons generated by a linear accelerator (Clinac 4/100, Varian, Palo Alto, CA, USA) or exposed to cisplatin (2 µg/ml, 1 h), cetuximab (Erbitux®; 30 nM, Merck KGaA, Darmstadt, Germany), gefitinib (Iressa®, 50 nM, AstraZeneca, London, UK) or dasatinib (Sprycel®, 10 nM, Bristol-Myers Squibb, New York, NY, USA). The effect of the treatments was evaluated after another 9 days in culture using the crystal violet assay described above.

### Cell cycle analysis

48 h after cell sorting by FACS, cells of the three subpopulations were detached using 0.25% trypsin and 0.02% EDTA, washed and resuspended in propidium iodide (PI) solution (0.4 mg/ml PI, 1 mg/ml sodium citrate, 60 μg/ml trizma base, 1.7 mg/ml spermine tetrahydrochloride and 0.01% nonidet P40; all from Sigma). Samples were analyzed by flow cytometry and cell cycle analysis was performed using ModFit (Verity Software House, Topsham, ME, USA).

### Plating efficiency assay

Immediately after cell sorting by FACS, cells were seeded into 6-well plates and after 10 days in culture the cells were fixed in 4% PFA and stained with crystal violet (0.04% in 1% ethanol). The number of colonies containing at least 32 cells was counted in a light microscope.

### Immunofluorescence staining

The epithelial phenotype was verified by immunofluorescence staining of cytokeratins as previously described [25]. Cells grown on glass coverslips were fixed in 4% PFA, incubated with 0.1% saponin and 5% fetal calf serum in PBS-A followed by incubation in a primary antibody towards pan-cytokeratin (1∶50; Novocastra Laboratories Ltd., Newcastle, UK) in 0.1% saponin and 5% fetal calf serum in PBS-A overnight. As secondary antibody a goat anti-mouse AlexaFluor 488 conjugated antibody (1∶400; Molecular probes; Invitrogen, Paisley, UK) was used. Cells were mounted in Vectashield medium (Vector Laboratories, Burlingame, CA, USA) and examined in an Olympus Vanox AHBS3 fluorescence microscope. Images were collected using an Olympus DP50 camera.

### Custom RT^2^ Profiler™ PCR array

The expression of 87 genes primarily associated with EMT, stemness, and apoptosis was analyzed using a custom-made PCR array plate from SABiosciences (Frederick, MD, USA). The PCR reaction was performed according to the manufacturer's protocol.

Total RNA was extracted with the RNeasy Mini Kit (Qiagen, Solna, Sweden) and cDNA was acquired using the RT^2^ First Strand Kit (SABiosciences). DNA and RNA contamination was assessed using a genomic DNA control and a positive PCR control, respectively. The average of five housekeeping genes (beta-2-microglobulin [B2M], hypoxanthine phosphribosyltransferase 1 [HPRT1], ribosomal protein L13a [RPL13A], glyceraldehyde-3-phosphate dehydrogenase [GAPDH], and beta-actin [ACTB]) was used to normalize the Ct-values. Data were calculated using the comparative Ct method to present the data as a fold difference in expression level relative to a control sample [26].

### Quantitative real-time PCR (qPCR)

Total RNA was extracted with the RNeasy Mini Kit (Qiagen, Solna, Sweden) or High Pure RNA Isolation Kit (Roche, Mannheim, Germany), and cDNA was obtained using High Capacity RNA-to-cDNA Kit (Applied Biosystems, Stockholm, Sweden). The mRNA expression of CDH1, CDH2, NANOG, SOX1, TWIST1, FOXC2, FN1 and MMP7 was analyzed using a 7 500 Fast Real-Time PCR system and FAM/MGB probes (Applied Biosystems). All reactions were performed according to the manufacturer's instructions. GAPDH was amplified as an internal standard and the data were calculated using the comparative Ct method.

### Western blot analysis

Aliquots of 50 µg protein were subjected to western blot analysis as described elsewhere [27]. The antibodies used were: mouse anti-NANOG (1∶100; Santa Cruz Biotechnology, Santa Cruz, CA, USA), mouse anti-SOX1 (1∶10000; R&D Systems, Minneapolis, MN, USA), mouse anti-vimentin (1∶200; Santa Cruz), rabbit anti-N-cadherin (1∶1000; Abcam), rabbit anti-fibronectin1 (1∶200; Sigma-Aldrich, St Louis, MO, USA), mouse anti-CD44 (1∶1000; Cell Signaling, Danvers, MA, USA) and rabbit anti-EGFR (1∶1000; Santa Cruz) followed by a HRP (horse radish peroxidase) conjugated goat anti-rabbit antibody (1∶1500; Santa Cruz) or a HRP conjugated goat anti-mouse antibody (1∶1500; Santa Cruz). Equal loading was verified by reprobing the membranes with a HRP-conjugated anti-actin antibody (1∶2000; Santa Cruz).

### Statistical analysis

Data was statistically evaluated using one-way ANOVA followed by Bonferroni's multiple comparison test. The level of significance was set to p≤0.05.

## Results

### Identification of subpopulations of cells in HNSCC cell lines

**Figure 1:**
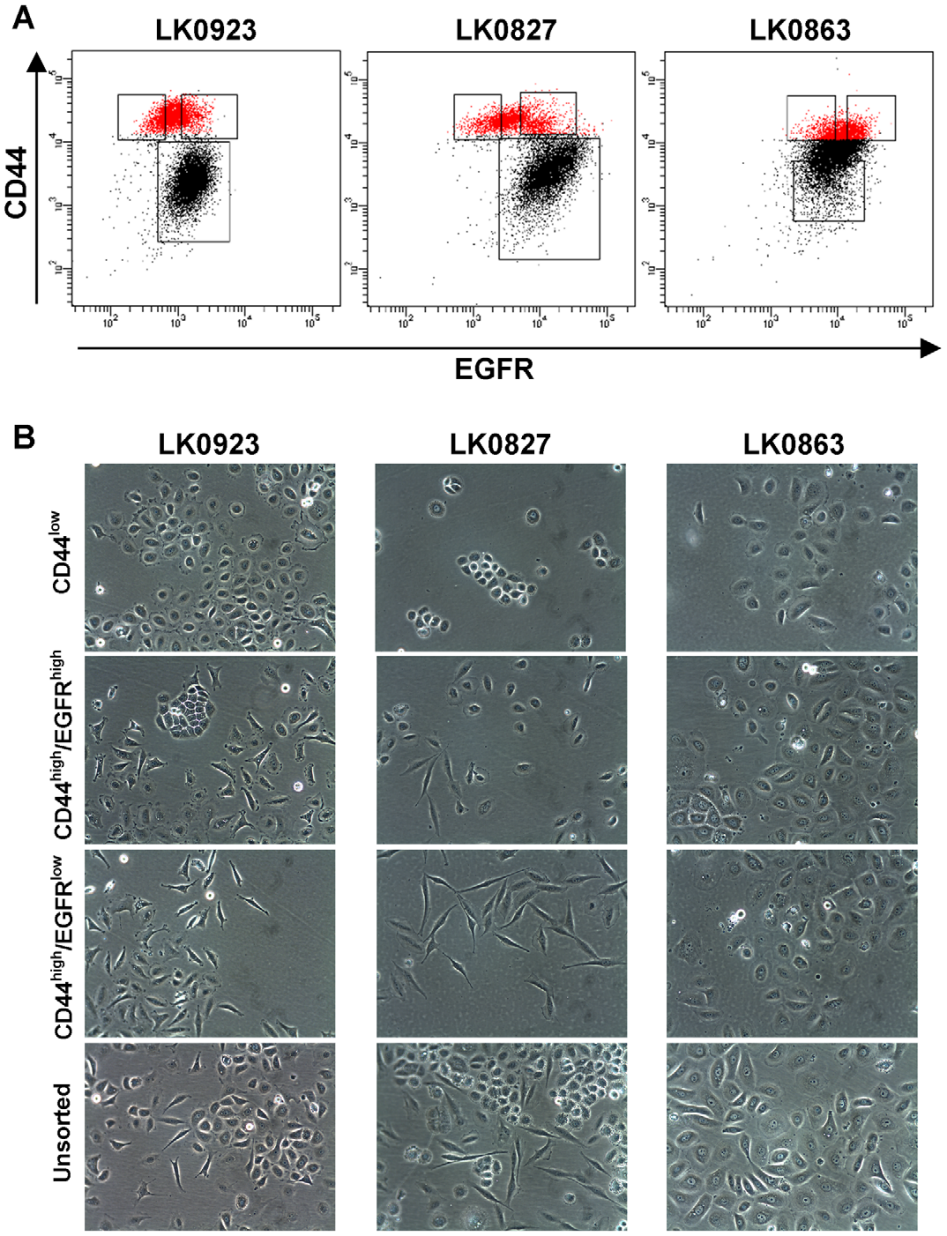
**A:** Identification of subpopulations of cells in HNSCC cell lines. Dot plot analysis of LK0923, LK0827 and LK0863 cells co-stained with an APC-conjugated anti-CD44 antibody and a PE-conjugated anti-epidermal growth factor receptor (EGFR) antibody. The red colored areas represent the CD44^high^ cells, and the three boxes display the gating of the different subpopulations from which cells were isolated for further experiments. **B:** Light microscope images of CD44^low^, CD44^high^/EGFR^high^ and CD44^high^/EGFR^low^ populations (3 days after sorting) and unsorted LK0923, LK0827 and LK0863 cell cultures.

In order to identify subpopulations with different phenotypes, the surface expression of CD44 was investigated in three HNSCC cell lines (LK0923, LK0827 and LK0863) by flow cytometry. In the LK0923 and LK0827 cell lines, two distinct subgroups of cells, having either high or low CD44 cell surface expression, were identified; however, no such populations could be observed in the LK0863 cell line (Figure 1A). The CD44^low^ subgroup was more abundant as compared to the CD44^high^ subgroup (72.3%±9.1 vs 23.6%±9.2 in LK0923; 68.9%±9.6 vs 27.6±9.3 in LK0827).

As lack of EGFR cell surface expression has been associated with stemness [19], cells were co-stained with anti-CD44 and anti-EGFR antibodies after which the CD44^high^ and CD44^low^ populations were analyzed with respect to their EGFR expression. Interestingly, a part of the LK0923 and LK0827 CD44^high^ population showed a lower EGFR expression compare to cells in the CD44^low^ population (Figure 1A). Thus, the CD44^high^ subgroup was further divided into two groups, based on their EGFR expression, resulting in three subpopulations; CD44^low^, CD44^high^/EGFR^high^ and CD44^high^/EGFR^low^. These subpopulations, defined by the gating in Figure 1A, were collected by FACS for further analysis. The proportion of each sorted population in each cell line is described in Table S1. In the LK0923 and LK0827 cell lines, the three populations differed from each other with regard to cell morphology (Figure 1B). While cells of the CD44^high^/EGFR^low^ population showed a spindle-shaped EMT-like morphology, the CD44^low^ population was dominated by cobblestone-shaped cells. Consequently, the CD44^high^/EGFR^high^ population contained both cobblestone- and spindle-shaped cells. In the LK0863 cell line, in which no distinct populations was observed based on CD44 surface expression, cells of the CD44^low^, CD44^high^/EGFR^high^ and CD44^high^/EGFR^low^ populations did not differ from each other morphologically (Figure 1B).

### Characterization of the CD44^low^, CD44^high^/EGFR^high^ and CD44^high^/EGFR^low^ subpopulations

**Figure 2:**
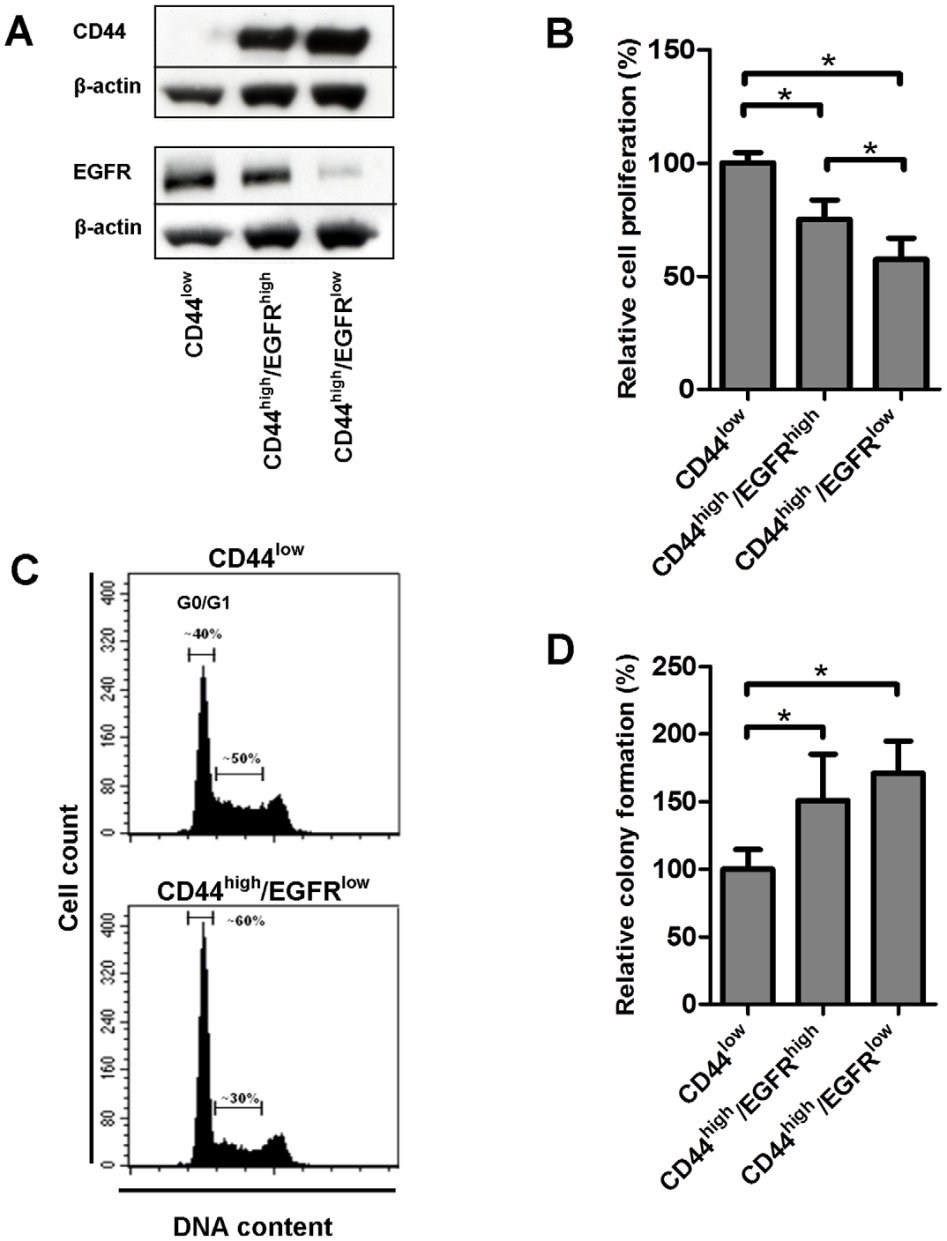
Characterization of the CD44^low^, CD44^high^/EGFR^high^ and CD44^high^/EGFR^low^ subpopulations within the LK0923 cell line. **A:** Expression of CD44 and epidermal growth factor receptor (EGFR) in sorted subpopulations (β-actin was used as a loading control). **B:** Proliferation rate of subpopulations. Data represents mean ± SD from three experiments in triplicate, *p≤0.05. **C:** Cell cycle distribution comparison between CD44^low^ and CD44^high^/EGFR^low^ cells. Data from one representative experiment out of two are shown. **D:** Plating efficiency, as assessed by colony formation in sorted subpopulations. Data represents mean ± SD from three experiments in duplicate, *p≤0.05.

To further characterize sorted populations the LK0923 cell line, which displayed the most distinct CD44^high^ population, was used. The total EGFR and CD44 expression of the three subgroups was analyzed by western blot. CD44^high^/EGFR^low^ cells had a very low expression of total EGFR as compared to the other two subgroups and in the CD44^low^ population no CD44 expression was detected (Figure 2A).

To study the epithelial phenotype of the sorted populations an immunofluorescence staining of pan-cytokeratin was performed. This analysis showed a stronger staining in the CD44^low^ cells as compared to the two CD44^high^ populations six days after isolation, but after an additional 30 days in culture the difference was not as prominent, indicating that the two most EMT-like populations changed into cells with a more epithelial phenotype with time (Figure S1).

Furthermore, sorted populations were cultured for 30 days and thereafter analyzed with regard to the heterogeneity of the culture. Cultures originating from the CD44^high^/EGFR^high^ population consisted of 51% CD44^low^ and 42% CD44^high^ cells while the CD44^high^/EGFR^low^ population consisted of 10% CD44^low^ and 82 % CD44^high^ cells. In contrast, the CD44^low^ population contained only CD44^low^ cells 30 days after sorting. Taken together, both the CD44^high^/EGFR^high^ and CD44^high^/EGFR^low^ populations had the capacity to recapitulate the heterogeneity of the culture but to a varying degree.

Next, the proliferation rate and cell cycle distribution of the three subpopulations were investigated. Cells of the CD44^high^ population were found to have a lower proliferation rate as compared to CD44^low^ cells (Figure 2B). Furthermore, there were a higher number of cells in the G0/G1 phase within the CD44^high^/EGFR^low^ population as compared to the CD44^low^ population (60% and 40%, respectively; Figure 2C).

To evaluate the plating efficiency of the three investigated subpopulations the cells were seeded at low density and after 10 days the number of colonies formed was determined. The CD44^low^ population showed a significant lower plating efficiency when compared to the other two subpopulations (Figure 2D).

Taken together, the CD44^high^/EGFR^low^ population consists of more cells in G0/G1, resulting in a lower proliferation rate, but has a higher plating efficiency and probably a higher clonogenic potential.

### The CD44^high^/EGFR^low^ population shows upregulation of EMT-associated genes

In order to screen for differences between sorted subpopulations within the LK0923 cell line the expression of a selection of genes involved in stemness, EMT and apoptosis was investigated using a real-time PCR array. To analyse the differences between the subgroups a hierarchical cluster analysis was performed (TIGR Multiexperiment viewer 4) which revealed differences in 28 genes (Figure S2). The greatest difference in gene expression was found between the CD44^high^/EGFR^low^ and the CD44^low^ subpopulations, while the CD44^high^/EGFR^high^ cells, in concordance with the morphological analysis, showed similarities with both of the other two subgroups. When comparing the CD44^high^/EGFR^low^ population to the CD44^low^ population, differences in mRNA level could be observed in 27 genes, 20 of which were upregulated and 7 downregulated (Table 1). Of these 27 differentially expressed genes 14 can be related to an EMT phenotype (VIM, MMP2, MMP7, TIMP1, TWIST1, COL3A1, CDH1, CDH2, ITGA5, FN1, FOXC2, WNT5A, WNT5B and SPARC). Interestingly, CD44^high^/EGFR^low^ cells also showed an upregulation of two stem cell genes, NANOG and SOX1.

**Table 1:** Differentially expressed genes in LK0923 subpopulations.

	Fold difference*	
	*CD44^high^/EGFR^high^*	*CD44^high^/EGFR^low^*
**Upregulated**		
NANOG (nanog homebox)	1.4	2.3
BMP3 (bone morphogenetic protein 3)	0.89	4.2
FGF2 (fibroblast growth factor 2)	4.9	5.8
FGF3 (fibroblast growth factor 3)	-	3.7
FGF4 (fibroblast growth factor 4)	0.87	5.9
SOX1 (sex determining region Y box 1)	1.4	2.3
VIM (vimentin)	81.3	93.7
MMP2 (matrix metalloproteinase-2)	17.8	24.4
MMP7 (matrix metalloproteinase-7)	3.2	6.5
TIMP1 (tissue inhibitor of metalloproteinase-1)	1.9	3.1
TWIST1 (twist homolog 1)	2	2.3
COL3A1 (collagen type III. alpha 1)	271	3125
CDH2 (N-cadherin)	6.3	6.5
ITGA5 (integrin. alpha 5)	3	3.2
FN1 (fibronectin 1)	9.7	19.2
FOXC2 (forkhead box C2)	4.6	7.1
EGF (epidermal growth factor)	0.85	3.5
WNT5a (wingless-type MMTV integration site family. type 5a)	10.3	49.9
WNT5b (wingless-type MMTV integration site family. type 5b)	2.5	3.4
SPARC (secreted protein. acidic. cysteine rich)	4.2	6.1
**Downregulated**		
BMP7 (bone morphogenetic protein 7)	0.01	0.007
FOS (FBJ murine osteosarcoma viral oncogene homolog)	0.19	0.1
CDH1 (E-cadherin)	0.12	0.01
ERBB3 (V-erb-b2 erythroblastic leukemia viral oncogene homolog 3)	0.14	0.05
AREG (amphiregulin)	0.26	0.05
TGFA (transforming growth factor alpha)	0.47	0.14
ALDH1A1 (Aldehyde dehydrogenase 1 family. member A1	0.17	0.16

* =  Fold difference in gene expression relative to the CD44^low^ population.

**Figure 3:**
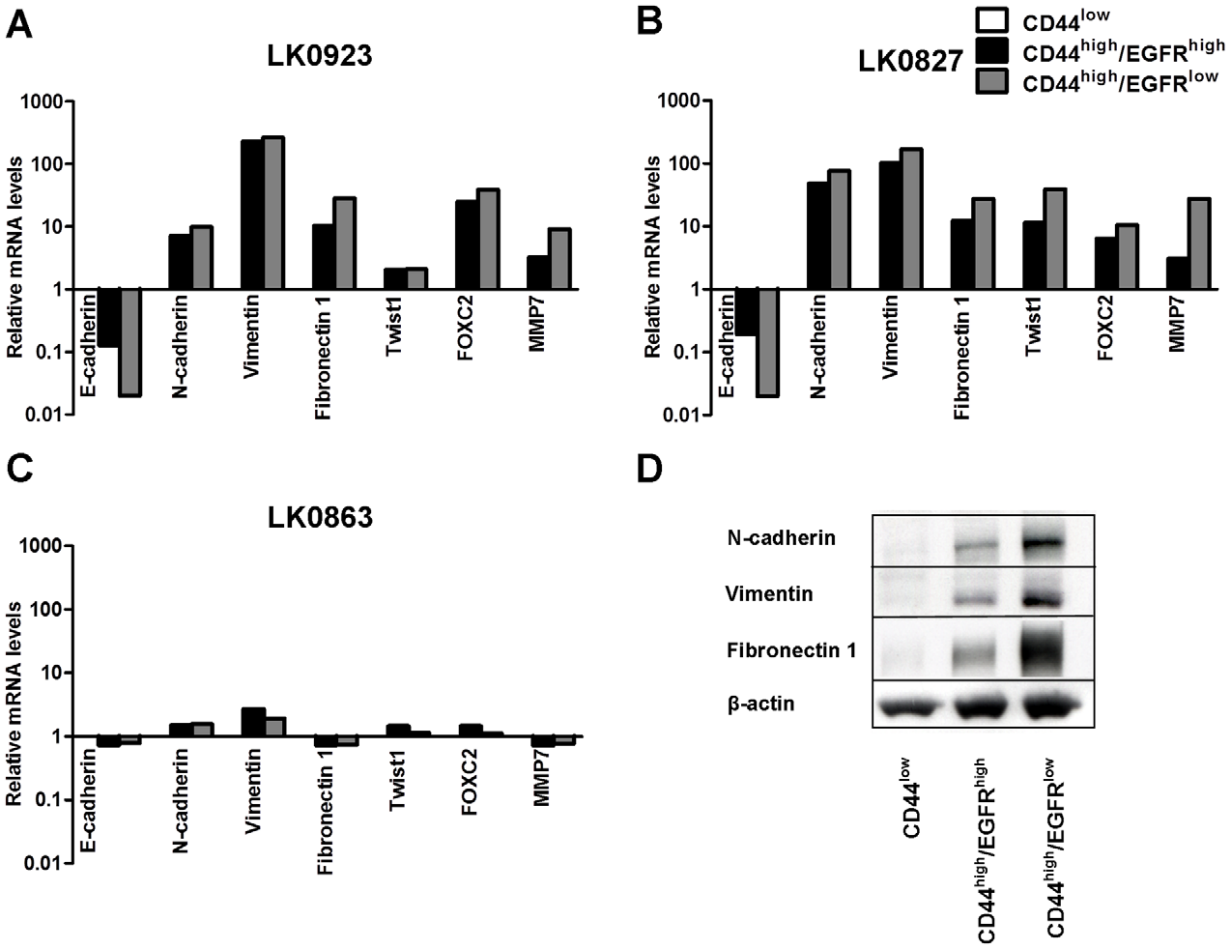
The CD44^high^ population shows upregulation of EMT-associated genes. **A–C:** Relative mRNA expression levels of epithelial-mesenchymal transition-associated genes; E-cadherin, N-cadherin, vimentin, fibronectin1, Twist1, FOXC2 and matrix metalloproteinase 7 (MMP7) in (**A**) LK0923, (**B**) LK0827 and (**C**) LK0863 subpopulations. The expression levels in the CD44^high^/EGFR^high^ and CD44^high^/EGFR^low^ populations are displayed here as fold difference relative to the CD44^low^ population (GAPDH was used as an internal standard). **D:** Western blot analysis of N-cadherin, vimentin and fibronectin1 in LK0923 populations (β-actin was used as loading control).

The real-time PCR array data was verified by analyzing the mRNA expression of CDH1 (E-cadherin), CDH2 (N-cadherin), VIM (vimentin), FN1 (fibronectin 1), TWIST1, FOXC2 and MMP7 by qPCR (Figure 3A). The mRNA expression level of the above mentioned factors was also determined in LK0827 and LK0863 populations. Similar to what was found in LK0923, the CD44^high^/EGFR^high^ and CD44^high^/EGFR^low^ populations of the LK0827 cell line showed a distinct EMT phenotype (Figure 3B). On the other hand, in the LK0863 cell line no differences in mRNA expression were observed between sorted populations (Figure 3C). Moreover, the difference in expression of N-cadherin, vimentin and fibronectin 1 was verified at the protein level in LK0923 by western blot analysis (Figure 3D).

**Figure 4:**
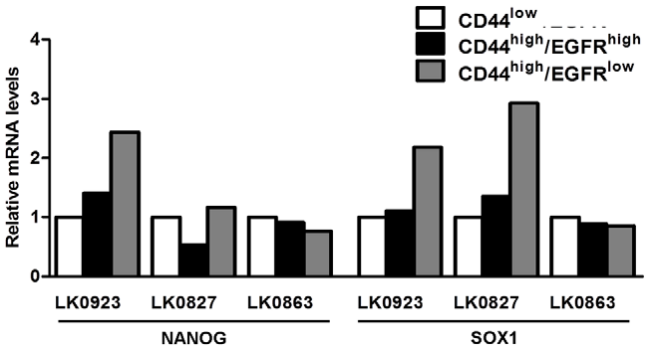
The CD44^high^/EGFR^low^ population shows upregulation of cancer stem cell-associated genes. Relative mRNA expression levels of the stemness genes NANOG and SOX1 in LK0923, LK0827 and LK0863 subpopulations, here displayed as fold difference relative to the CD44^low^ population (GAPDH was used as an internal standard).

The upregulation of the CSC markers NANOG (LK0923) and SOX1 (LK0923 and LK0827) in CD44^high^/EGFR^low^ cells was confirmed at the mRNA level (Figure 4), but could not be verified at the protein level (data not shown).

### Sorted subpopulations show differences in treatment sensitivity

To evaluate if differences in treatment sensitivity exist between the three subpopulations, sorted cells were exposed to ionizing γ-irradiation (4 Gy), cisplatin (2 μg/ml, 1 h), cetuximab (30 nM), gefitinib (50 nM) or dasatinib (10 nM).

**Figure 5:**
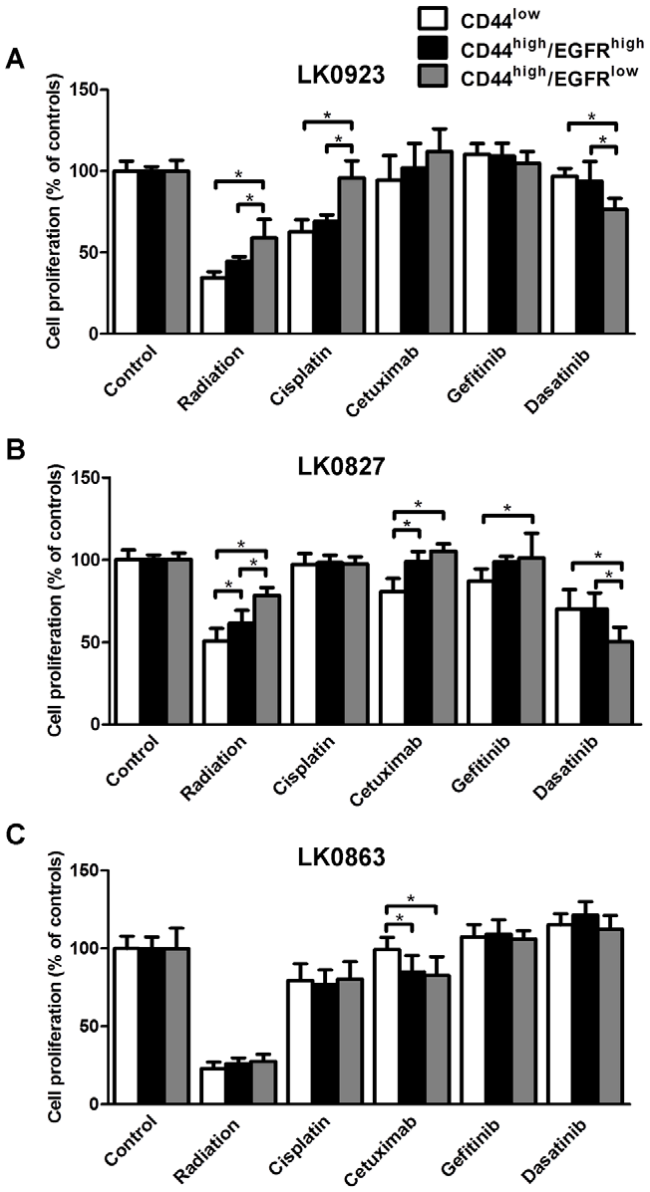
Sorted subpopulations show differences in treatment sensitivity. Treatment sensitivity of CD44^low^, CD44^high^/EGFR^high^ and CD44^high^/EGFR^low^ cells, isolated from (**A**) LK0923, (**B**) LK0827 or (**C**) LK0863, and exposed to radiation (4 Gy), cisplatin (2 μg/ml, 1h), cetuximab (30 nM), gefitinib (50 nM) or dasatinib (10 nM). Data represents mean ± SD from three experiments in triplicate, *p≤0.05.

The LK0923 CD44^high^/EGFR^low^ cells proved to be highly resistant to cisplatin treatment when compared to both CD44^low^ and CD44^high^/EGFR^high^ cells (Figure 5A). The CD44^high^/EGFR^low^ population of LK0827 and LK0923 cells displayed a significantly higher resistance against radiotherapy compared to the other two subpopulations whereas in LK0863 no differences were found (Figures 5A, 5B and 5C). Furthermore, when cetuximab was added to CD44^high^/EGFR^low^ cells from LK0827 and LK0923 they proved to be resistant. In LK0923 no differences between subpopulations were observed after exposure to gefitinib, but in LK0827 the CD44^low^ cells were significantly more sensitive to gefitinib compared to CD44^high^/EGFR^low^ cells.

Taken together, in general the LK0827 and LK0923 CD44^high^/EGFR^low^ cells showed a low sensitivity to radiation, cisplatin, cetuximab and gefitinib relative to their CD44^high^/EGFR^high^ and CD44^low^ counterparts (Figure 5A and 5B). On the other hand, they were found to be more sensitive to treatment with the multi-BCR/ABL and Src family tyrosine kinase inhibitor dasatinib.

## Discussion

In the present study we investigated the possibility of using the surface expression of EGFR and CD44 to find populations of cells within HNSCC cell lines that differ from each other with respect to phenotype and treatment sensitivity. Three populations, consisting of CD44^low^, CD44^high^/EGFR^high^ and CD44^high^/EGFR^low^ cells, respectively, were analyzed after cell sorting by FACS. The CD44^high^/EGFR^low^ population harbors a larger number of cells in G0/G1, shows a lower proliferation rate and a higher plating efficiency as compared to the CD44^high^/EGFR^high^ and CD44^low^ populations. In line with these results, Le Roy et al showed a connection between normal and cancer keratinocyte fate and asymmetric distribution of EGFR during mitosis. Moreover, they identified three populations in normal and cancer keratinocyte cultures: a small but constant pool of Rh123^−^ (Rhodamine 123, a marker of side population) and EGFR^−^ quiescent cells, a small but variable in size between normal and cancer cell cultures pool of Rh123^−^ /EGFR^+^ (perhaps activated stem cells), and a third major pool of Rh123^+^ /EGFR^+^ transient amplifying cells. The Rh123^−^ /EGFR^−^ culture consisted of more cells in G0/G1 and cells with higher clonogenic ability [19].

In two out of three investigated cell lines, CD44^high^/EGFR^low^ cells displayed a distinct EMT-like phenotype with spindle-shaped morphology, as well as upregulation of EMT markers. However, no apparent CSC phenotype could be observed in these cells. These results suggest that the surface expression of CD44 (high) and EGFR (low) identify a population of cells with an EMT phenotype. This population is enriched with CSCs; however, additional markers are required for specific selection of CSCs.

Recently, Biddle *et al* provided evidence that within HNSCC tumors two distinct CSC phenotypes exist, one CD44^high^/epithelial specific antigen (ESA)^high^ and one CD44^high^/ESA^low^ [28]. The CD44^high^/ESA^low^ population shows an EMT-phenotype and seems to have properties similar to the CD44^high^/EGFR^low^ population described in this study. Both CSC populations described by Biddle *et al* could switch their epithelial or mesenchymal traits to reconstitute the cellular heterogeneity but the CD44^high^/ESA^low^ population to less extent (contains 50% of bipotent cells compared to 100% bipotent cells in the CD44^high^/ESA^high^ population). In line with their results, 30 days after sorting CD44^high^/EGFR^high^ cells display a greater capacity to reconstitute the cellular heterogeneity as compared to CD44^high^/EGFR^low^ cells.

The connection between CSCs and EMT has become more evident over the last couple of years. In 2008, Mani et al described that induction of EMT in mammary epithelial cells resulted in cells gaining stem cell properties [29], a phenomena also seen in pancreatic cancer cells [30]. In HNSCC it has been shown that cells obtained from spheroid cultures, which demonstrated stem cell properties, also had an EMT phenotype with elevated vimentin and α-smooth muscle actin levels [31].

The importance of EMT in treatment resistance has recently been targeted for investigation in different types of cancers [32]. In both pancreatic cancer and HNSCC it has been shown that cells with an EMT phenotype are more resistant to cisplatin treatment [7], [33], and the same pattern was observed when studying the response to radiation [34], [35] and cetuximab [35]. The subpopulations within the LK0923 and LK0827 cell lines differed with regard to treatment response. The CD44^high^/EGFR^low^ cells displayed a significantly lower sensitivity to cisplatin (LK0923) and radiotherapy (LK0923 and LK0827) when compared to the CD44^low^ and CD44^high^/EGFR^high^ cells. In one of our previous studies, in which radioresistant and radiosensitive HNSCC cell lines were compared by microarray analysis, many of the identified key regulator genes were associated with EMT [36]. One of these key regulators, FN1, was significantly upregulated in radioresistant tumor cells both at the mRNA and protein level. In a similar study, comparing cisplatin resistant and cisplatin sensitive HNSCC cell lines, MMP7 was shown to be a biomarker for cisplatin resistance [37]. In line with these data, the treatment resistant CD44^high^/EGFR^low^ population within the LK0923 and LK0827 cell lines showed increased levels of both FN1 and MMP-7. A connection between EMT and upregulation of MMP-7 expression has earlier been described. For example, in A431 human SCC cells growing in 3D, an increase in MMP-7 expression was accompanied by a reduction of E-cadherin at the cell surface [38]. Moreover, in oral SCC cells cytoplasmic accumulation of β-catenin was found to induce Tcf/Lef-mediated transcriptional activity, upregulation of MMP-7 and induction of EMT [39].

In HNSCC it has been shown that an EMT phenotype correlates to resistance to cetuximab and radiation [35] and EMT-related changes in gene expression, e. g., downregulation of E-cadherin and upregulation of vimentin have been proposed as potential biomarkers for prediction of response after treatment with cetuximab and radiation [8]. Significant differences in treatment response to EGFR targeted therapies (cetuximab and gefitinib) could be detected between populations of LK0827 and a tendency (cetuximab) in LK0923. Notably, the CD44^high^/EGFR^low^ cells were more sensitive than the other two populations to the multi-BCR/ABL and Src family tyrosine kinase inhibitor dasatinib. These results indicate that cells with an EMT phenotype are more dependent on cell signaling mediated by these kinases. These results are in line with the findings from Mandal et al who described that HNSCC cells with an EMT phenotype had a higher level of Src activation, and that cells treated with a Src inhibitor showed an upregulation of E-cadherin [40]. Furthermore, in breast cancer it has been shown that cells with a high expression of vimentin are highly sensitive to dasatinib [41].

In conclusion, we here show for the first time that the combination of CD44 and EGFR cell surface expression can be used to identify a subpopulation of HNSCC cells that exhibit an EMT phenotype and resistance to radiotherapy, cisplatin and EGFR-targeted therapies. Interestingly, cells within this subpopulation show a high sensitivity to the multi-BCR/ABL and Src family tyrosine kinase inhibitor dasatinib, indicating that addition of dasatinib to conventional treatment could improve the clinical outcome in these patients.

## Supporting Information

Figure S1 **Cytokeratin distribution in sorted subpopulations within the LK0923 cell line.** Representative photographs showing immunofluorescent staining of pan-cytokeratin in CD44^low^, CD44^high^/EGFR^high^ and CD44^high^/EGFR^low^ populations six or 36 days after sorting.
(TIF)

Figure S2 **A hierarchical cluster analysis showing differences in gene expression levels between CD44^low^, CD44^high^/EGFR^high^ and CD44^high^/EGFR^low^ populations.** The mRNA expression of genes involved in stemness, EMT and apoptosis was investigated in subpopulations within the LK0923 cell line using quantitative PCR. A hierarchical cluster analysis was performed (TIGR Multiexperiment viewer 4) and the color code represents the normalized Ct values.
(TIF)

Table S1 **The proportions of gated populations in LK0923, LK0827 and LK0863.**
(DOCX)
