# Supplementary figures and images for: A CD44high/EGFRlow Subpopulation within Head and Neck Cancer Cell Lines Shows an Epithelial-Mesenchymal Transition Phenotype and Resistance to Treatment
Source: PLoS One. 2012 Sep 25;7(9):e44071. doi: 10.1371/journal.pone.0044071 (PMC3458050; doi:10.1371/journal.pone.0044071)

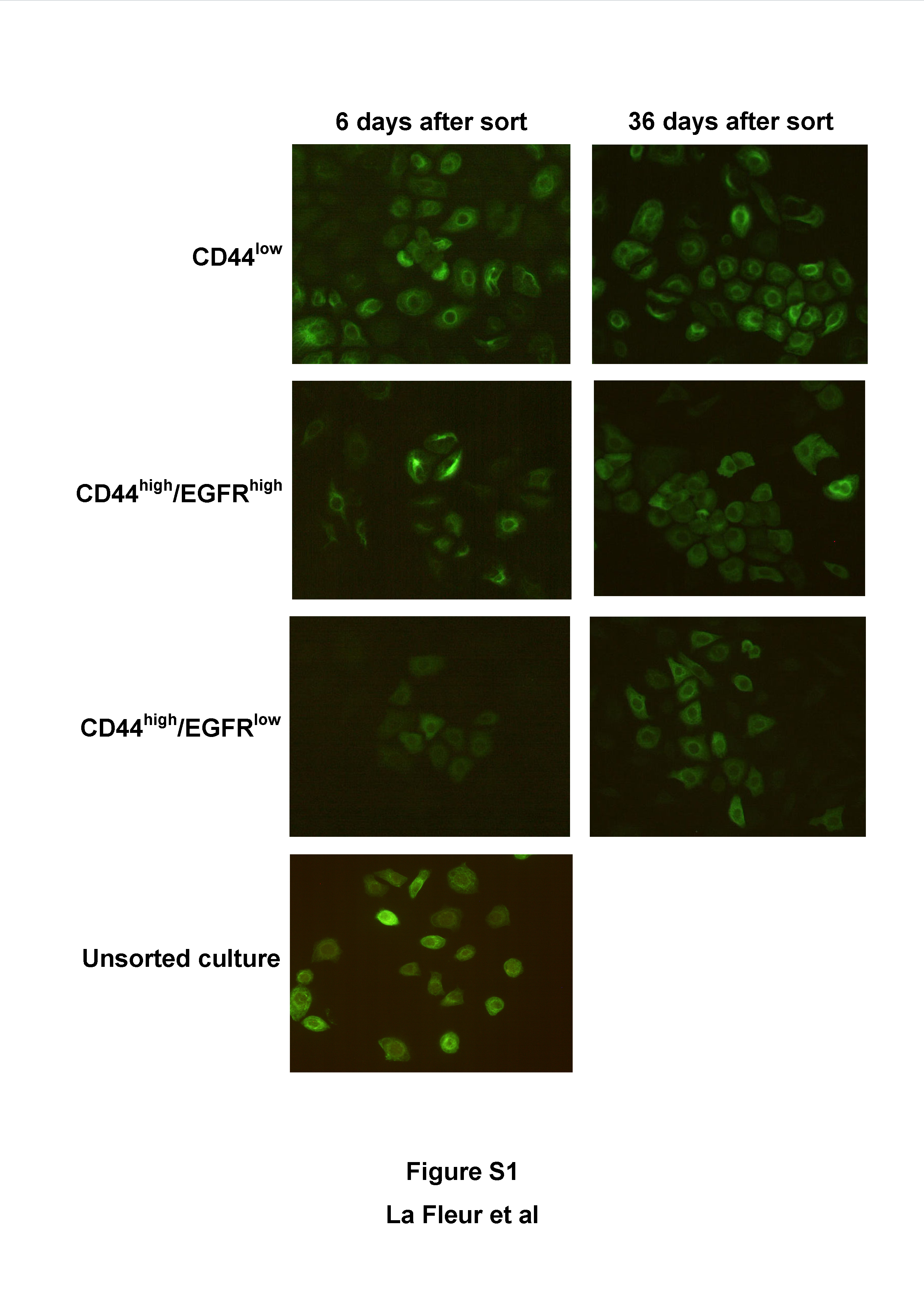

Supplement: Figure S1 — Cytokeratin distribution in sorted subpopulations within the LK0923 cell line. Representative photographs showing immunofluorescent staining of pan-cytokeratin in CD44low, CD44high/EGFRhigh and CD44high/EGFRlow populations six or 36 days after sorting. (TIF) [file pone.0044071.s001.tif]

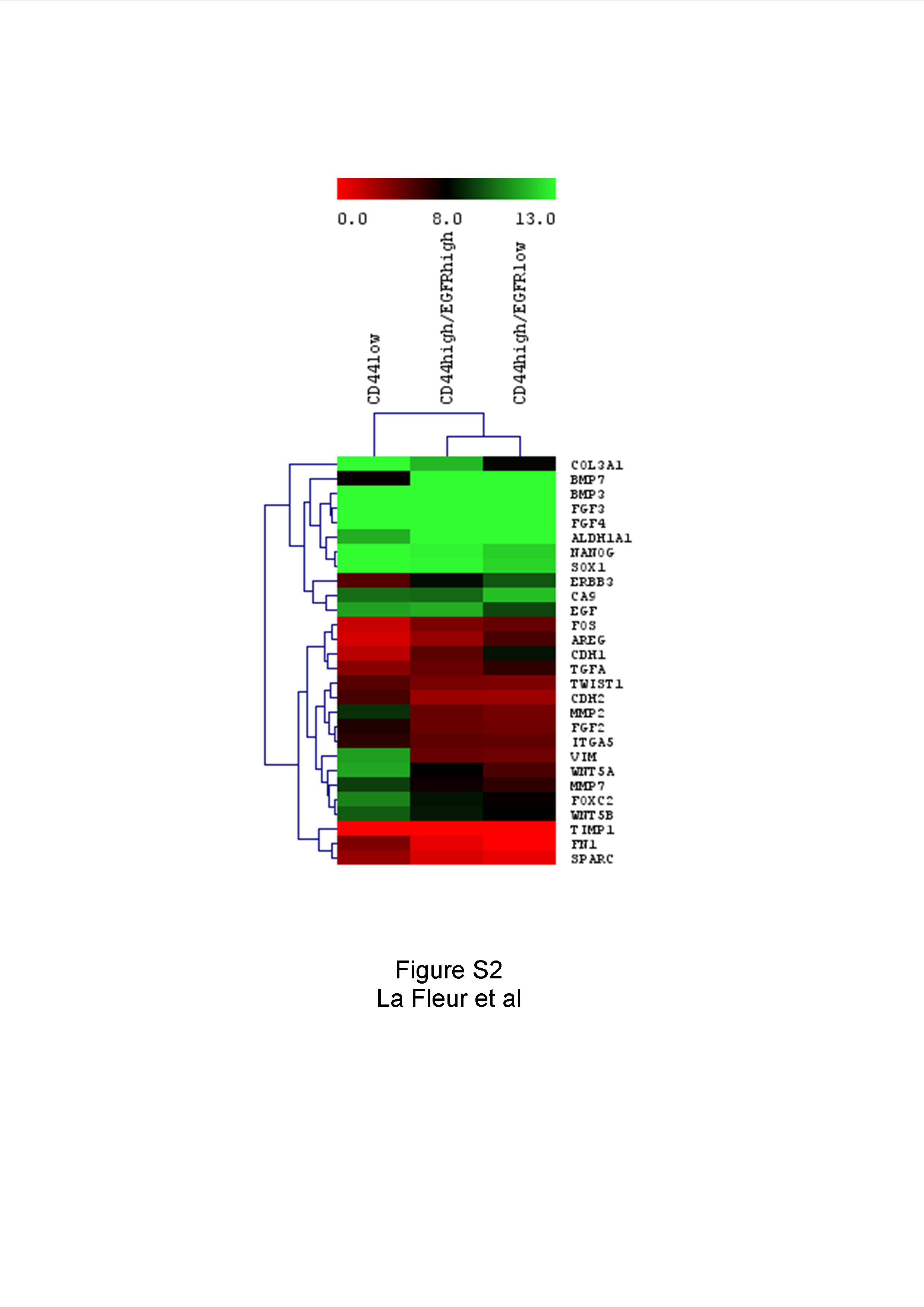

Supplement: Figure S2 — A hierarchical cluster analysis showing differences in gene expression levels between CD44low, CD44high/EGFRhigh and CD44high/EGFRlow populations. The mRNA expression of genes involved in stemness, EMT and apoptosis was investigated in subpopulations within the LK0923 cell line using quantitative PCR. A hierarchical cluster analysis was performed (TIGR Multiexperiment viewer 4) and the color code represents the normalized Ct values. (TIF) [file pone.0044071.s002.tif]
