# Supplementary material for: A CD44high/EGFRlow Subpopulation within Head and Neck Cancer Cell Lines Shows an Epithelial-Mesenchymal Transition Phenotype and Resistance to Treatment
Source: PLoS One. 2012 Sep 25;7(9):e44071. doi: 10.1371/journal.pone.0044071 (PMC3458050; doi:10.1371/journal.pone.0044071)
Supplement: Table S1 — The proportions of gated populations in LK0923, LK0827 and LK0863. (DOCX) [file pone.0044071.s003.docx]

| **Table S1** |  |  |  |
| --- | --- | --- | --- |
|  | **Gated populations, %** | | |
|  | *LK0923* | *LK0827* | *LK0863* |
| **CD44^low^** | 72,07 % ± 11,26 | 68,96 % ± 9,57 | 10,08 % ± 0,82 |
| **CD44^high^/EGFR^high^** | 8,98 % ± 3.09 | 9,31 % ± 3,33 | 12,42 % ± 3,57 |
| **CD44^high^/EGFR^low^** | 6,44 % ± 4,23 | 11,61 % ± 2,65 | 10,82 % ± 2,49 |
